# Psychological Processes Associated With Resilience in UK-Based Unpaid Caregivers During the COVID-19 Pandemic

**DOI:** 10.32872/cpe.10313

**Published:** 2022-12-22

**Authors:** Emma Wilson, Juliana Onwumere, Colette Hirsch

**Affiliations:** 1 Department of Psychology, Institute of Psychiatry, Psychology and Neuroscience, King’s College London, London, United Kingdom; 2 Health Service and Population Research, Institute of Psychiatry, Psychology and Neuroscience, King’s College London, London, United Kingdom; 3 ESRC Centre for Society and Mental Health, King’s College London, London, United Kingdom; 4 South London and Maudsley NHS Foundation Trust, London, United Kingdom; Philipps-University of Marburg, Marburg, Germany

**Keywords:** resilience, interpretation bias, emotion regulation, informal carers, unpaid caregivers, COVID-19

## Abstract

**Background:**

Unpaid caregivers have faced and dealt with additional challenges during the COVID-19 pandemic. Understanding the psychological processes associated with their resilience is warranted. The objective of this study was to examine the associations between resilience with mental distress, emotion regulation strategies (i.e., reappraisal and suppression) and interpretation bias in adult caregivers.

**Method:**

Participants were living in the UK, aged 18+, and consisted of 182 unpaid caregivers of an adult aged 18+ living with a long-term health condition, and 120 non-caregivers. Data were collected in an online study during the first national UK COVID-19 lockdown (May and September 2020). Hierarchical multiple regression analyses explored whether emotion regulation strategies and interpretation bias explained unique variance in levels of resilience in caregivers whilst controlling for anxiety and depression.

**Results:**

Compared to non-caregivers, caregivers reported higher levels of anxiety, depression, negative interpretation bias and lower levels of resilience. Emotion regulation strategies did not differ between groups. Within caregivers, greater resilience was associated with lower mood disturbance, a positive interpretation bias, and greater use of cognitive reappraisal and lower use of suppression strategies to regulate emotions. Emotion regulation and interpretation bias together predicted an additional 15% of variance in current levels of resilience.

**Conclusion:**

Our findings indicate that psychological mechanisms such as emotion regulation strategies, particularly reappraisal, and interpretation bias are associated with resilience in caregivers. Although preliminary, our findings speak to exciting clinical possibilities that could form the target of interventions to improve resilience and lower mental distress in unpaid caregivers.

## Highlights

- Negative interpretation bias, alongside use of emotional regulation strategies (i.e., suppression; reappraisal), and their association with resilience was investigated in unpaid caregivers for the first time.
- Caregivers report lower levels of resilience and higher levels of anxiety and depression compared to non-caregivers during the COVID-19 pandemic.
- The tendency to interpret information in more positive ways, and to use reappraisal as a way to regulate emotions, were associated with greater resilience in caregivers.
- Interpretation bias and reappraisal could form the target of future caregiver tailored interventions to improve resilience.

Data suggests the United Kingdom (UK) is facing an increase in negative mental health outcomes due to the impact of the COVID-19 pandemic (Li & Wang, 2020). Unpaid caregivers (also called informal carers, herein ‘caregivers’) have been defined as ‘anyone, including children and adults who looks after a family member, partner or friend who needs help because of their illness, frailty, disability, a mental health problem or an addiction and cannot cope without their support’ (NHS England, 2014). Pre-pandemic, caregivers represented around 7% of the UK population (Department for Work and Pensions, 2020) and Carers UK (2020) has suggested that numbers doubled from 6.5 million to 13.6 million during the COVID-19 pandemic. Compared to the general population and pre-pandemic, caregivers were at greater risk of anxiety and depression and poorer health outcomes (Smith et al., 2014). This is observable across different illness groups; for example, when caring for someone with dementia (Papadopoulos et al., 2019), cancer (LeSeure & Chongkham-ang, 2015), multiple sclerosis (McKeown et al., 2003), and a mental health condition (Young et al., 2019).

On 23^rd^ March 2020, the UK government introduced a nationwide lockdown with measures aimed to restrict transmission of the virus and mitigate pressure on the National Health Service (NHS). Measures included staying at home with few exceptions (e.g., essential purposes), working from home unless designated a ‘key worker’ and always maintaining social distancing rules. People in at-risk groups were asked to ‘shield’ by remaining indoors. Caregivers had to navigate the changes to their own routine and consider their own pre-existing health conditions and life situation (Onwumere, 2021; Vahia et al., 2020). Hence, in a group already at a heightened risk of social isolation (Hayes et al., 2015) and lower life satisfaction compared to non-caregivers (Naef et al., 2017), distress was exacerbated by social distancing rules and inability to access support from friends and family or formal services in their caring role (Baker & Clark, 2020; Whitley et al., 2021). Understanding how the psychological wellbeing of caregivers, relative to their non-caregiver peers, was impacted during the pandemic and the key mechanisms driving their presentations is an important step in informing future targeted interventions. However, these types of investigations have been limited. Nevertheless, emerging data suggests reduced psychological wellbeing (e.g., heightened anxiety/depressive symptoms, stress/distress related to caregiving, care burden) among family caregivers (Gallagher & Wetherell, 2020; Muldrew et al., 2022), although the psychological mechanisms driving these mood states remain less researched in the literature.

One psychological factor associated with better psychological functioning (i.e., positive adaptation) is resilience (Luthar et al., 2015; Seery et al., 2010), commonly defined as the ability to bounce back from adversity (Rutter, 1985, 1987; Southwick et al., 2015). This psychological process can fluctuate over time and across contexts, so one person may be resilient to certain adversities but not others (Egeland et al., 1993; Pooley & Cohen, 2010). Windle and Bennett’s (2012) theoretical resilience framework for caregivers also highlights how resilience is influenced by interactions in the environment and draws on social resources. Restricted access to important resources in health and social care during periods of lockdown, combined with the threat from the virus to the most vulnerable, may have impacted caregivers in particular, threatening their capacity to remain resilient. Identifying factors that may foster lower levels of distress and higher levels of resilience in caregivers during times of extra stress, such as a pandemic, could help us identify those who are likely to need extra support and better tailor future interventions; particularly when resources are limited (Rapado-Castro & Arango, 2021).

Resilience is associated with higher quality of life, better regulation of emotions, more positive emotions, and less perceived stress, anxiety and depression (Balmer et al., 2014; Troy & Mauss, 2011). In caregivers, reduced mood disturbance (e.g., lower levels of anxiety and depression) is recorded in those reporting higher levels of resilience (Simpson et al., 2015). Moreover, systematic review data suggests that higher resilience levels are linked to reductions in the risk of stress and care burden and supports greater role adaptation (Palacio González et al., 2020). To determine whether caregiver and non-caregiver populations in the UK differed in levels of resilience during early stages (first 3 months) of a global pandemic, data were collected using a widely used, multidimensional self-report measure of resilience with good psychometric properties (Connor & Davidson, 2003; Pangallo et al., 2015).

Given the potential importance of resilience to caregiver wellbeing and outcomes, it would seem important to also identify modifiable psychological mechanisms that can foster resilience, such as emotion regulation approaches (Palacio González et al., 2020). Common approaches include cognitive reappraisal (occurs before an emotion is experienced; seeking alternative perspectives in situations that may change the emotional response) and suppression (purposively attempting to suppress expressive behaviour while emotionally aroused, such as trying not to display anger or annoyance; Gross, 1998; Gross, 2014; Gross & Levenson, 1993). Reappraisal is seen as an opportunity to grow in times of adversity by reducing maladaptive appraisals (e.g., self-blame), whereas suppression involves the avoidance of expressing one’s feelings and may lead to negative outcomes (Gross & John, 2003; John & Gross, 2004). The links between emotional regulation and resilience are yet to be explored despite a hypothesised relevance between two concepts that are arguably connected (Kay, 2016). The limited work in this area has suggested that high levels of cognitive reappraisal may serve as a protective factor that fosters resilience after adverse situations (Polizzi & Lynn, 2021; Troy & Mauss; 2011), while expressive suppression may have a negative effect on resilience (Hong et al., 2018; Mouatsou & Koutra, 2021).

Another psychological mechanism that might potentially expand our understanding of resilience in caregivers is interpretation bias, which is the tendency to draw negative conclusions from ambiguous information (Hirsch et al., 2016). There is already data to suggest that lower levels of interpretation bias are linked to greater resilience in groups such as women living beyond breast cancer (Booth et al., 2022; Gordon et al., 2022) and in adolescents (Booth et al., 2022). Such findings support a cognitive model of psychological resilience (Booth et al., 2022), whereby interpretation bias influences levels of resilience and is a key mechanism for maintaining internalising disorders such as mood conditions. Moreover, interpretation biases may interfere with certain protective emotion regulation strategies (e.g., reappraisal), impacting the regulation of negative affect (Joormann & Siemer, 2011). It was therefore anticipated that cognitive reappraisal would be associated with interpretation bias, and suppression associated with more negative interpretation biases of ambiguous situations. Given the challenges faced by unpaid caregivers, it is important to explore the relevance to their wellbeing of these potentially modifiable psychological mechanisms and by doing so, potentially inform the development of targeted and care focused support interventions.

## Study Aims

First, we sought to examine caregivers reports of depression, anxiety and resilience, alongside their levels of negative interpretations and compare these to non-caregiver populations. Second, we wanted to assess whether more negative interpretations and suppression of emotions, as well as less use of reappraisal, are associated with, and help predict, resilience levels in UK caregivers between May to September 2020 of the COVID-19 pandemic.

### Hypothesis 1

Caregivers compared to non-caregivers will have lower levels of resilience, and higher levels of anxiety and depression. Exploratory analysis will see if negative interpretation bias, emotion regulation (reappraisal and suppression) varies between caregivers and non-caregivers.

### Hypothesis 2

Within the caregiver population, greater resilience will be associated with lower levels of negative interpretation bias and expressive suppression, and greater use of cognitive reappraisal.

### Hypothesis 3

Within the caregiver population, emotion regulation and interpretation bias will contribute extra and unique variance in levels of resilience in a model which controls for factors known to be associated with resilience – anxiety and depression.

## Method

### Participants

Participants were aged 18+ and living in the UK. We recruited 182 caregivers and 120 non-caregivers. Caregivers could participate if they were not in a paid caring role (except for any state benefits/financial support for carers), had been in a caring role for 6 months or more, for someone aged 18+ who has a long-term condition commonly associated with caregiving (i.e., dementia, cancer, multiple sclerosis, and any mental health condition). Participants were recruited through social media, online message boards, charities (e.g., webpages or newsletters), the Join Dementia Research forum and Call for Participants.

### Materials and Measures

#### Demographic Questions

Participants completed several demographic questions regarding age, ethnicity, gender, employment status and relationship status. Questions were completed about their experience of the pandemic, including whether they believed they had had COVID-19, were currently self-isolating/quarantining (i.e., not leaving the house or having visitors), and whether they were a paid keyworker (i.e., paid workers in certain key sectors defined as critical to the COVID-19 response; Department for Education, 2021).

Caregivers were asked additional questions about the people they provide care for (i.e., number they care for, their relationship to them, their condition). If caregivers selected more than one medical condition, caregivers were asked to stipulate whether it was the primary condition of the person they care for. For caregivers caring for more than one person, they were asked to respond in relation to the person they currently spent most time caring for. Questions covered specific diagnosis, gender, age, employment status of the person cared for, estimated number of hours spent in this caregiving role per week, whether they live together and duration of their caring role. Caregivers were also asked if they had people to confide in and if so, how many. See Supplementary Materials 1 for full list of questions.

#### Questionnaire Measures

##### Connor-Davidson Resilience Scale (CD-RISC)

This 25-item questionnaire (Connor & Davidson, 2003) measures resilience over the past month on a 5-point Likert scale (1 = *not at all* to 5 = *true nearly all the time*). Total scores range from 0 – 100 with higher scores reflecting greater resilience. Example item: *‘I tend to bounce back after illness, injury, or other hardships’.* The CD-RISC has demonstrated high internal consistency in previous studies with caregivers of older adults (α = .94; Ong et al., 2018), people with dementia (α = .89; Ruisoto et al., 2020), and severe mental illness (α = .93; Mulud & McCarthy, 2017). Present sample Cronbach’s α = .91.

##### Generalized Anxiety Disorder 7 (GAD-7)

This 7-item questionnaire (Spitzer et al., 2006) measures symptoms of anxiety over the past 2 weeks and asks participants ‘how often have you been bothered by the following problems?’ on a 4-point Likert scale (1 = *not at all* to 4 = *never*). A sum score is calculated, and scores assigned to the following categories of anxiety: Minimal (< 4), mild (5-9), moderate (10-14), severe (15-21). Example item: *‘worrying too much about different things’*. The GAD-7 has been found to have high/good internal reliability in the general population (Löwe et al., 2008) and in carers (α = .93; Lappalainen et al., 2021). Present sample Cronbach’s α = .91.

##### Patient Health Questionnaire 9 (PHQ-9)

This 9-item questionnaire (Kroenke & Spitzer, 2002) measures symptoms of depression over the past 2 weeks and asks participants ‘how often have you been bothered by the following problems?’ on a 4-point Likert scale (1 = *not at all* to 4 = *never*). The sum of scores indicates the following depression severities: None (<4), mild (5-9), moderate (10-14), moderately severe (15-19), severe (20-27). Example item: *‘little interest or pleasure in doing things’*. The PHQ-9 has been found to be a valid and reliable measure of depression (Kroenke et al., 2001) and is widely used in caregiver studies (Kishita et al., 2020; Ping Pang et al., 2020). Present sample Cronbach’s α = .91.

##### Emotion Regulation Questionnaire (ERQ)

This 10-item questionnaire (Gross & John, 2003) measures how individuals use two emotional regulation strategies in daily life: cognitive reappraisal and expressive suppression. The reappraisal scale contains six items (e.g., ‘*when I’m faced with a stressful situation, I make myself think about it in a way that helps me stay calm’*) and suppression contains four items (e.g., *‘I control my emotions by not expressing them’*), using 7-point Likert scales (1 = *strongly disagree* to 7 = *strongly agree*). The score for each subscale is the mean of the items (range 1 – 7) and the ERQ has been used in carer populations (α range from .67 to .84; Aerts et al., 2019; Lamothe et al., 2018). Present sample Cronbach’s α = .74.

#### Interpretation Bias Task

##### Scrambled Sentences Test (SST)

Adapted from Wenzlaff and Bates (1998, 2000) and used in Hirsch et al. (2020); in 20 trials, participants select 5 words from 6 randomly presented words to form a grammatically correct sentence. Potential completions are positive or negative interpretations of self-referent statements. The task is completed over five minutes while holding a six-digit string in mind. The digit string has been used previously to add a cognitive load, allowing latent biases to be observed and limit participants from guessing the purpose of the sentence scrambling task, reducing the risk of answers being subject to demand characteristics such as social desirability (Krahé et al., 2022; Schoth & Liossi, 2017). An interpretation bias score is created by dividing the number of grammatically correct positively unscrambled sentences by the number of correct negatively unscrambled sentences. Index scores range from 0 to 1, with higher scores denoting a more positive interpretation bias.

### Procedure

The survey was hosted on Qualtrics with all data collected between May and September 2020, between the middle of the first COVID-19 lockdown and the start of the UK home nations gradually reopening. Both caregiver and non-caregiver groups completed the same core survey (questionnaires, SST), and caregivers completed additional demographic questions about the person(s) they care for. The survey took 35 – 50 minutes to complete and participants could enter a prize draw for Amazon vouchers: 1 of 20 £10 prizes, 1 of 2 £50 prizes, or 1 of 2 £100 prizes. The study was approved by the King’s College London Research Ethics Committee (approval number: HR-19/20-14617) and participants provided consent and data electronically.

### Statistical Analysis

Bivariate descriptive statistics were used to describe sample characteristics and summarise scores of study measures. Continuous variables were expressed as means (standard deviation, SD). Two-tailed *t*-tests for continuous variables (e.g., age) and chi-squared tests for categorical variables (e.g., gender) were used to test for group differences in sociodemographic factors and study variables (H1). Effect sizes were calculated using Cohen’s *d* for *t*-tests, and Phi and Cramer’s V for chi-squared tests. Associations between study variables in caregivers were quantified using Pearson’s correlation coefficient (H2). In the caregiver sample, a hierarchical regression tested the hypothesis that emotion regulation strategies (i.e., reappraisal and suppression) and interpretation bias would contribute significant variance, beyond anxiety and depression, in predicting levels of resilience (H3). Anxiety and depression were entered as independent variables in the model’s first step. Emotion regulation and interpretation bias were entered into the second step as independent variables. Resilience was the outcome variable. Statistical significance was set at *p* < .05. SPSS versions 26 and 27 were used to conduct all analyses.

## Results

See Table 1 for participant demographics and Table 2 for characteristics of the individuals that caregivers were caring for and their caregiving role. Participants were predominantly women and White British, with a higher proportion in the caregiver group. The higher rates of women as caregivers is similar to levels reported in the literature (Tur-Sinai et al., 2020). Other demographic characteristics were well-matched. Caregivers most often cared for someone with dementia (66%) and lived with the person they cared for (61%). Mental health conditions included depression (*n* = 8), anxiety (*n* = 4), psychosis/schizophrenia, (*n =* 3), PTSD (*n* = 2), bipolar disorder (*n* = 2), personality disorder (*n* = 2), eating disorder (*n* = 2), OCD (*n* = 1), other/multiple conditions including autism and learning difficulties (*n* = 12), not reported (*n* = 8).

**Table 1:** Demographic Characteristics

Baseline Characteristic	Caregiver sample (*n* = 182)	Non-caregiver sample (*n* = 120)	Statistical test, significance value and effect size
	*n* (%)	*n* (%)	
Age – *M* (*SD*)^a^	56.36 (13.48)	53.76 (17.65)	*t* (207.98) = 1.37, *p* = .172, *d* = .166
Ethnicity			Non-White British vs. White British, χ^2^(1) = 7.64, *p* = .006, φ = -.159
Arab	–	1 (0.8)	
Bangladeshi	1 (0.5)	–	
Black British	3 (1.6)	–	
Chinese	1 (0.5)	1 (0.8)	
Indian	3 (1.6)	1 (0.8)	
Pakistani	1 (0.5)	–	
Other	1 (0.5)	22 (18.3)	
White and Asian	1 (0.5)	1 (0.8)	
White and Black Caribbean	1 (0.5)	1 (0.8)	
White British	159 (87.4)	90 (75.0)	
White Gypsy or Irish Traveller	1 (0.5)	–	
White Irish	5 (2.7)	3 (2.5)	
Gender^b^			χ^2^ (1) = 12.19, *p* = .001, φ = .201
Woman	155 (85.2)	82 (68.3)	
Man	26 (14.3)	37 (30.8)	
Employment status			χ^2^ (3) = 1.68, *p* = .641, V = .075
Full-time employment	25 (13.7)	23 (19.2)	
Part-time employment	34 (18.7)	22 (18.3)	
Retired	62 (34.1)	39 (32.5)	
Other	61 (33.5)	36 (30.0)	
Relationship status			χ^2^ (3) = 11.15, *p* = .011, V = .192
Married/ domestic partnership	108 (59.3)	49 (40.8)	
Cohabiting	23 (12.6)	18 (15.0)	
Single	26 (14.3)	31 (25.8)	
Separated, divorced, widowed	25 (13.7)	22 (18.3)	
COVID-19 questions			
Caregiver has had COVID-19^c^	25 (13.7)	19 (15.8)	χ^2^ (1) = 0.96, *p* = .327, φ = -.063
Self-isolating/ in quarantine^de^	20 (11.0)	18 (15.1)	χ^2^ (2) = 2.59, *p* =.274, V = .093
Considered a ‘key worker’^fg^	36 (19.8)	22 (18.3)	χ^2^ (1) = .08, *p* = .781, φ = .016

^a^Declined to say*: n* = 1. ^b^Other: *n* = 2. ^c^Respondents asked: *n* = 245. ^d^Declined to say*: n* = 1. ^e^By self-isolating/ in quarantine we mean not leaving the house for any reason and avoiding contact with anyone outside the household. ^f^Declined to say*: n* = 1. ^g^A ‘key worker’ was defined as someone who worked in: health and social care, education and childcare, key public services, local and national government, food and other necessary goods, public safety and national security, transport, utilities, communication and financial services. Phi (φ) and V (V) are measures of effect size for chi-square tests.

**Table 2:** Characteristics of the Person/People Caregivers Cared for and the Caregiving Role

Characteristics	Participants (*n* = 182)
**Number they care for, *mean (SD)***	1.25 (0.62)
Primary condition, *n* (%)^a^	
Dementia	120 (65.9)
Multiple sclerosis	8 (4.4)
Cancer	10 (5.5)
Mental health condition	44 (24.2)
Relationship, *n* (%)	
Spouse/partner	66 (36.3)
Son/daughter	62 (34.1)
Parents	34 (18.7)
Other relative/friend/neighbour	20 (11.0)
Hours per week in caregiving role, *n* (%)	
0 – 19	60 (33.0)
20 – 49	49 (26.9)
50 – 90	24 (13.2)
Over 100	49 (26.9)
Duration of caregiving role, *n* (%)	
Under 12 months	18 (9.9)
1 – 5 years	75 (41.2)
5 – 10 years	45 (24.7)
Over 10 years	44 (24.2)
Live with person cared for, *n* (%)	
Yes	111 (61.0)
No	71 (39.0)
Has someone to confide in, *n* (%)	136 (74.7)
Number of confidents, *mean* (*SD*)	3.32 (2.51)

^a^If more than one condition listed, participant asked to provide primary condition of person they care for.

Several post hoc power analyses were conducted to test for the power of the analyses conducted for each of our hypotheses (e.g., *t*-test, correlation, multiple regression). Except for two *t*-tests with small effect sizes (i.e., ERQ-R, ERQ-S; see Table 3), the minimum power achieved for all analyses was .82.

**Table 3:** Scores for Questionnaires and Interpretation Bias Measure, by Group

Measures	Caregiver group (*n* = 182)	Non-caregiver group (*n* = 120)	*t*-test and significance value
	*M* (*SD*)	*M* (*SD*)	
Questionnaire			
Resilience (CD-RISC)	62.21 (13.86)	66.98 (12.58)	*t* (300) = -3.04, *p* = .003, *d* = 0.36
Anxiety (GAD-7)	6.91 (5.44)	4.03 (4.63)	*t* (281.09)* = 4.92, *p* < .001, *d* = 0.57
Depression (PHQ-9)	8.95 (6.60)	4.63 (5.00)	*t* (294.30)* = 6.47, *p* < .001, *d* = 0.74
Emotion Reappraisal (ERQ-R)	4.44 (1.18)	4.62 (1.03)	*t* (300) = -1.33, *p* = .183, *d* = 0.16
Emotion Suppression (ERQ-S)	3.77 (1.35)	3.54 (1.23)	*t* (300) = 1.49, *p* = .137, *d* = 0.18
Interpretation bias (SST)	0.67 (0.24)	0.76 (0.20)	*t* (285.26)* = -3.60, *p* < .001, *d* = 0.42

*Note.* CD-RISC = Connor-Davidson Resilience Scale; GAD-7 = Generalised Anxiety Disorder Questionnaire; PHQ-9 = Patient Health Questionnaire; ERQ-R = Emotion Regulation Questionnaire – Reappraisal; ERQ-R = Emotion Regulation Questionnaire – Suppression; SST = Scrambled Sentences Test.

*Equal variances not assumed.

### Do Caregivers Exhibit Lower Levels of Resilience and Higher Levels of Distress Than Non-Caregivers and Is Interpretation Bias More Negative in Caregivers?

The mean scores for all questionnaires are presented in Table 3. In keeping with Hypothesis 1, caregivers demonstrated lower levels of resilience, higher levels of anxiety, depression and interpretation bias with small to medium effect sizes (*d* = 0.36 to 0.74). Exploratory analysis found that emotion regulation techniques did not differ significantly between groups.

### Is There an Association Between Resilience, Emotion Regulation Techniques and Interpretation Bias in Caregivers?

To examine how resilience may be associated with emotion regulation techniques and more negative interpretations (H2), we conducted Pearson’s correlations; see Table 4 (non-caregiver sample on request). As expected, caregivers reporting greater resilience had a more positive interpretation bias, and greater use of cognitive reappraisal and lower use of suppression strategies to regulate emotions. Furthermore, greater resilience was associated with lower levels of anxiety and depression symptoms.

**Table 4:** Correlations Between Resilience, Anxiety, Depression, Emotion Regulation and an Interpretation Bias Measure (SST) in Caregiver Participants

Measure	1	2	3	4	5
1. CD-RISC					
2. GAD-7	-.50***				
3. PHQ-9	-.57***	.80***			
4. ERQ-R	.49***	-.31***	-.31***		
5. ERQ-S	-.21**	.23***	.293**	-.03	
6. SST	.64***	-.65***	-.75***	.41***	-.26***

*Note*. *n* = 182; CD-RISC = Connor-Davidson Resilience Scale; GAD-7 = Generalised Anxiety Disorder Questionnaire-7; PHQ-9 = Patient Health Questionnaire-9; ERQ-R = Emotion Regulation Questionnaire – Reappraisal; ERQ-S = Emotion Regulation Questionnaire – Suppression; SST = Scrambled Sentences Test.

**p < .01. ***p < .001.

To determine whether emotion regulation and/or interpretation bias helps account for levels of resilience, we conducted a hierarchical multiple regression (see Table 5). In Step 1, processes known to be covariates of resilience were entered: anxiety and depression. In Step 2 emotion regulation via reappraisal, emotion regulation via suppression and interpretation bias scores were entered into the model. In Step 1, the model accounted for 33% of the variance in resilience, *F*(2, 179) = 44.69, *p* < .001 (see Table 5). When emotion regulation techniques and interpretation bias were added in Step 2, an additional 15% of variance of resilience was explained (Adjusted *R*^2^ = .48), *F*(5, 176) = 33.96, *p* < .001. Furthermore, both interpretation bias (β = .35, *p* < .001) and cognitive reappraisal (β = .28, *p* < .001) significantly predicted independent variance in resilience, but not emotion regulation via suppression (β = -.05, *p* = .385). Results did not change when other covariates associated with caregiving were added into the model (i.e., gender, age, ethnicity, time caring per week, duration of caregiving role; see Supplementary Analyses 2).

**Table 5:** Hierarchical Regression Analysis Testing the Influence of our Predictors on Resilience

Predictor variable	*B*	*SE*	β	*t*
Step one				
GAD-7	-0.33	0.26	-.13	-1.28
PHQ-9	-0.98	0.21	-.47	-4.59***
Step two				
GAD-7	-0.10	0.23	-.04	-0.42
PHQ-9	-0.37	0.22	-.18	-1.69
ERQ-R	3.26	0.69	.28	4.71***
ERQ-S	-0.51	0.58	-.05	-0.87
SST	20.35	4.90	.35	4.15***

*Note. n* = 182. *B* = unstandardized coefficient; *SE =* standard error; β = standardised coefficient; GAD-7 = Generalised Anxiety Disorder Questionnaire; PHQ-9 = Patient Health Questionnaire; ERQ-R = Emotion Regulation Questionnaire – Reappraisal; ERQ-R = Emotion Regulation Questionnaire – Suppression; SST = Scrambled Sentences Test.

***p < .001.

## Discussion

This study aimed to investigate reported levels of resilience and wellbeing in unpaid adult caregivers of a person aged 18+ with a long-term condition (specifically, multiple sclerosis, dementia, any mental health condition, and/or cancer) compared to non-caregivers during a period of additional stress – the COVID-19 pandemic – and what role, if any, potentially modifiable psychological mechanisms (i.e., interpretation bias, emotion regulation via reappraisal and suppression) had on carers’ reported levels of resilience. To the best of our knowledge, this represents the first investigation of its kind.

As predicted and in keeping with non-pandemic data, caregivers reported lower levels of resilience and greater levels of depression and anxiety compared to non-caregivers (our control condition). Our pattern and direction of findings for these higher levels of caregiver emotional distress and lower resilience support published findings using samples from before (Onwumere et al., 2017; Smith et al., 2014; Windle & Bennett, 2012) and during the pandemic (Kalb et al., 2021).

Our study confirmed for the first time that caregivers’ resilience levels were associated with greater levels of positive interpretation bias, greater levels of reappraisal emotion regulation techniques and, to a lesser extent, lower levels of suppression. A more positive interpretation bias as well as greater use of cognitive reappraisal accounted for an additional 15% of the variance in resilience scores, with interpretation bias and use of reappraisal to regulate emotions both accounting for independent variance in resilience. To support a more nuanced understanding of these findings, an investigation with a similar sample outside of a global pandemic would be indicated.

Cognitive reappraisal and expressive suppression are independent constructs within the area of emotion regulation (Moore et al., 2008). Reappraisal is central to managing one’s emotional reaction to stressful situations, encouraging positive outcomes over time and important for understanding resilience, whereas suppression fails to address the emotion internally (Troy & Mauss, 2011). Although both forms were associated with resilience, the current data found reappraisal, a cognitive construct, more relevant to fostering resilience than suppression, a non-cognitive construct that is focused on changing only the outward expression of emotions (Gross, 2014). This supports recent literature, which has found more mixed findings for the relation between expressive suppression and resilience, suggesting that situational factors may influence the longer-term adaptive or maladaptive role of suppression (Polizzi & Lynn, 2021). As a first step, supporting caregivers with emotional reappraisal techniques may be more beneficial than targeting expressive suppression.

Our findings on interpretation bias add to a growing body of literature that explores the impact of this cognitive bias in other populations, including adolescents with eating disorders, individuals with anxiety disorders, pregnant women, parents and their offspring (Hirsch et al., 2021; Rowlands et al., 2020; Subar & Rozenman, 2021). All highlight the risk of negative outcomes for negative interpretation biases. Importantly, interpretation bias and reappraisal are known to be modifiable mechanisms that can be targeted in psychological interventions; fostering a more positive interpretation bias or facilitating greater use of reappraisal techniques to regulate emotions could be beneficial in increasing resilience in caregivers. Interventions to foster resilience both at an individual or familial level, and population level, are crucial for managing future pandemics and any longstanding negative impacts from COVID-19 (Ameis et al., 2020), as well as challenges associated with long-term caregiving in non-pandemic times.

It is notable that while resilience is lower in caregivers (62.21) than non-caregivers (66.98), scores are much lower than general populations prior to the COVID-19 pandemic (80.4; Connor & Davidson, 2003). Indeed, our caregiver sample have similar levels of resilience to patients commencing a trial for PTSD (62.0; Krystal et al., 2014) and psychiatric outpatients with a history of recent trauma (64.3; Glass et al., 2019), although not as severe as some other PTSD populations (e.g., 49.8 to 55.7; Davidson et al., 2006; McGuire et al., 2018). While the mean levels of anxiety and depression reported in caregivers fell within the non-clinical range (i.e., a score of 7 or below for the GAD-7 and 9 or below for the PHQ-9), levels were higher compared to non-caregivers (*p* < .001, *d* = 0.57 to 0.84) and 46.2% still reported clinical levels of anxiety and 25.8% reported clinical levels of depression. This remains consistent with current literature (Giebel et al., 2021; Li et al., 2021) and offers further support of the need to consider the wellbeing of caregivers.

The results offer early support for potential therapeutic avenues. Cognitive behaviour therapy (CBT), for example, fosters more positive interpretations by reducing maladaptive thinking (DeRubeis et al., 2008) and a greater use of reappraisal to regulate emotion (Smits et al., 2012). Another approach to increase positive interpretation bias is cognitive bias modification for interpretations (CBM-I); this involves repeated computerised practice in generating more positive interpretations (Menne-Lothmann et al., 2014). It is possible that a caregiver focused CBM-I intervention could be tailored to focus on promoting more positive interpretations of ambiguous and potentially negative situations that caregivers frequently encounter (e.g., uncertainty and ambiguity around implications for changes in symptoms in the person they care for). Future qualitative studies could explore the specific caregiver stressors contributing to negative interpretations and its sequalae, compared to those unrelated to caregiving, to see if there is a generalised or situation-specific bias.

There are limitations of the current study. Firstly, it is cross-sectional, with data collected data within four months near the start of the pandemic. It therefore does not provide information on trajectories of resilience over the longer term during the pandemic, nor provide information on the extent to which interpretation bias predicts later levels of resilience in the caregiver populations. Furthermore, we are unable to determine the extent to which general caregiver stress was exacerbated by the pandemic for a given individual in this sample due to lack of pre-pandemic data. While caregiving roles can be held by anyone, irrespective of demography, ethnic minority participants were largely underrepresented in our sample. This is important given that many of the key conditions in this study disproportionately affect some racial and ethnic minority groups, such as dementia, and caregiver experiences may differ across cultures (Liu et al., 2021). Consequently, the under-representation limits generalisability of findings to the wider population.

Additionally, our study did not look at the impact of looking after children during the pandemic. Managing home-schooling alongside other responsibilities such as work undoubtedly contributed to additional challenges. These have been considered in great depth elsewhere. Finally, participants could only be recruited and participate via the Internet and therefore less likely to represent the experiences of informal caregivers with no or limited access to the Internet, or those with less time to take part due to increase caregiving demands. In 2020, groups less likely to have internet access in the UK included the over 75s (46%), retired individuals (28.9%) and persons who self-assessed as having a disability (18.6%; Office for National Statistics, 2021).

As convenience samples, our groups were also not matched on all demographic variables. Specifically, control participants were more frequently European White, men and single, as compared to caregivers. The under representation of particular groups is part of a broader issue in UK health focused surveys (Harrison et al., 2020). Nevertheless, future studies should aim to better match the control group to the caregiver sample.

In summary, caregivers were reporting less resilience and higher levels of anxiety and depression compared to non-caregivers during the COVID-19 pandemic. Importantly, the tendency to interpret information in more positive ways and to use reappraisal as a way to regulate emotions was associated with greater resilience and could form the target of future caregiver interventions to improve resilience.

## Supplementary Materials

The Supplementary Materials contain the following items (for access see Index of Supplementary Materials below):

- *Supplementary Materials 1*: Additional questions asked to unpaid caregivers
- *Supplementary Materials 2*: Hierarchical regression analysis testing the influence of our predictors on resilience while controlling for additional covariates

WilsonE.
OnwumereJ.
HirschC.
 (2022). Supplementary materials to "Psychological processes associated with resilience in UK-based unpaid caregivers during the COVID-19 pandemic"
[Additional information]. PsychOpen. 10.23668/psycharchives.9297
PMC988112136762350
